# Efficiency of *Spirulina* sp. in the Treatment of Model Wastewater Containing Ni(II) and Pb(II)

**DOI:** 10.3390/ma18153639

**Published:** 2025-08-01

**Authors:** Eleonora Sočo, Andżelika Domoń, Mostafa Azizi, Dariusz Pająk, Bogumił Cieniek, Magdalena M. Michel, Dorota Papciak

**Affiliations:** 1 Department of Inorganic and Analytical Chemistry, Faculty of Chemistry, Rzeszow University of Technology, 35-959 Rzeszow, Poland; 2 Department of Water Purification and Protection, Faculty of Civil, Environmental Engineering and Architecture, Rzeszow University of Technology, 35-959 Rzeszow, Poland; dpapciak@prz.edu.pl; 3 Institute of Environmental Engineering, Warsaw University of Life Sciences-SGGW, 02-787 Warsaw, Poland; mostafa_azizi@sggw.edu.pl (M.A.); magdalena_michel@sggw.edu.pl (M.M.M.); 4 Department of Casting and Welding, Faculty of Mechanical Engineering and Aeronautics, Rzeszow University of Technology, 35-959 Rzeszow, Poland; pajak@prz.edu.pl; 5 Institute of Materials Engineering, College of Natural Sciences, University of Rzeszow, Pigonia 1, 35-310 Rzeszow, Poland; bcieniek@ur.edu.pl

**Keywords:** biosorbent, biosorption, nickel, lead, heavy metals, *Spirulina* sp., kinetic, isotherm

## Abstract

In this work, the biosorption potential of *Spirulina* sp. as an effective and eco-friendly biosorbent for the removal of Ni(II) and Pb(II) ions from aqueous solutions was investigated. Detailed characterization of the biosorbent was carried out, including surface morphology, chemical composition, particle size, zeta potential, crystallinity, zero-point charge, and functional group analysis. Batch tests were performed to determine the kinetic constants and adsorption equilibrium of the studied ions. The adsorption behavior of *Spirulina* sp. was described using six adsorption isotherms. The best fit was obtained for the Redlich-Peterson and Langmuir isotherms, indicating that monolayer adsorption occurred. The maximum biosorption capacities for Ni(II) and Pb(II) were 20.8 mg·g^−1^ and 93.5 mg·g^−1^, respectively, using a biosorbent dose of 10 g·L^−1^, initial metal concentrations ranging from 50 to 5000 mg·L^−1^, at pH 6, 20 °C, and a contact time of 120 min. Low values of the mean free energy of adsorption (E) in the Dubinin–Radushkevich and Temkin model (0.3 and 0.1 kJ·mol^−1^ for Pb(II) and 0.35 and 0.23 kJ·mol^−1^ for Ni(II)) indicate the dominance of physical processes in the ion binding mechanism. The adsorption of Pb(II) ions was more effective than that of Ni(II) ions across the entire range of tested concentrations. At low initial concentrations, the removal of Pb(II) reached 94%, while for Ni(II) it was 80%.

## 1. Introduction

Environmental pollution with heavy metals is one of the most serious challenges of contemporary environmental engineering. The ongoing industrialization contributes to the increase in the concentration of these elements in surface waters, which negatively affects their biological structure, the functioning of ecosystems, and the safety of using water resources [1,2,3,4,5,6]. Often, the average concentrations of heavy metals in surface water reservoirs significantly exceed the maximum permissible values for drinking water [7]. There are over 700 different chemical pollutants in water [8]. These include heavy metals, which are released into water every day from various natural sources (volcanic eruptions, weathering, soil erosion) and anthropogenic sources (industrial, mining, agricultural activities, uncontrolled waste disposal) [2,9,10,11,12]. Due to their properties, including biodegradability, bioaccumulation capacity, and toxicity at low concentrations, heavy metals pose a threat to both human health and the balance of ecosystems [11,13,14,15,16]. The group of heavy metals with the highest environmental and toxic risk includes chromium, nickel, copper, zinc, cadmium, lead, mercury, and arsenic [17,18]. Even in trace concentrations, these elements pose a serious threat—their presence in water can lead to growth and development disorders, hormonal changes (including feminization of men), and an increase in the incidence of waterborne diseases, affecting both developed and developing countries [19,20]. In order to minimize their impact on the environment, it is necessary to develop effective and ecological technologies for their removal.

Traditional methods of water purification from heavy metals, although effective, have significant limitations, including high costs, significant energy consumption, and generation of secondary pollutants [21,22]. In recent years, the industry has been paying increasing attention to techniques based on adsorption. This mechanism involves physicochemical binding of heavy metal ions to the surface of the adsorbent, mainly through ion exchange or formation of complexes with functional groups placed on the adsorption material. Additionally, raw materials of natural origin (e.g., peat, charcoal, clays), solid waste from industrial processes (e.g., fly ash, slag), agricultural by-products (e.g., rice bran, citrus peels, banana peels, coconut shells, nut shells), and biosorbents are increasingly considered as effective and inexpensive adsorbents [21,23,24,25]. Among the latter, microalgae seem to be particularly promising, as they effectively and selectively bind heavy metal ions due to their large specific surface area and the presence of various functional groups [26,27,28,29]. It is estimated that although the number of microalgae species ranges from about 30,000 to over a million, only fifteen of them are suitable for mass commercial cultivation [30]. *Spirulina* sp. stands out among them—its cells are rich in functional groups, i.e., carboxyl, amino, phosphate, hydroxyl, which facilitates the formation of stable complexes with metals. Additionally, *Spirulina* sp. is characterized by rapid growth, simplicity of cultivation, and fully biodegradable biomass, which makes it an ecological solution for water purification [31,32,33]. The use of microalgae in removing heavy metals is superior to traditional methods in terms of energy efficiency, operating costs, reduced sediment formation, reduced greenhouse gas emissions, and the possibility of recovering nutrients from biomass [34]. Materials based on Spirulina sp. have demonstrated high potential as effective adsorbents for removing various heavy metals [35,36,37,38,39,40,41,42]. Moreover, Spirulina sp. is also effective in the removal of dyes [43,44,45,46,47,48,49,50,51,52,53,54] and organic micropollutants [55,56,57,58,59,60]. The adsorption capacity of Spirulina sp. can vary significantly depending on the type of pollutant and experimental conditions (for example, for cadmium, it can reach values up to 625 mg·g^−1^ [61]). Such high capacities and efficiencies, obtained using different forms of Spirulina sp. (live, dead, dried, or modified), indicate its great flexibility and environmental friendliness. In connection with the above, Spirulina sp. can be considered as an attractive alternative to conventional adsorbents, such as activated carbon. However, it should be noted that its effectiveness depends to a large extent on the precise adjustment of the process conditions to the characteristics of a specific pollutant. This emphasizes the need to optimize the adsorption parameters to achieve the best results in practical applications.

Building on this promising background, the present work aimed to investigate the biosorption potential of *Spirulina* sp. biomass specifically for the removal of nickel(II) and lead(II) ions from aqueous solutions. The selection of Pb(II) and Ni(II) as target pollutants is justified by their widespread occurrence in industrial effluents as well as their significant toxicity and persistence in aquatic environments. In this study, the influence of key process parameters, such as the initial metal concentration and the adsorbent–adsorbate contact time, on adsorption efficiency was analyzed. Additionally, the biosorbent surface was characterized, and the adsorption mechanisms were explored using kinetic models and adsorption isotherms. The results obtained contribute to assessing the potential of Spirulina sp. as an alternative, environmentally friendly biosorbent for water purification from heavy metals.

## 2. Materials and Methods

### 2.1. Algae Biomass

The research used dried, powdered Spirulina sp. algae obtained from a commercial product commonly used as food for humans or animals (Bio Planet S.A.-Superfoods, Warsaw, Poland). Organic Spirulina is a type of blue-green microalga with a characteristic spiral shape. This freshwater alga, available in powdered form, is sourced from high-quality algae cultivated under controlled conditions. According to the manufacturer’s data, the Spirulina biomass contains approximately 60% protein, 5.9% fat (including 1.1% saturated fatty acids), 11% carbohydrates (including 3% sugars), 5.1% fiber, and 1.8% salt.

### 2.2. Characteristics of Algae Biomass

#### 2.2.1. Analysis of Surface Morphology and Chemical Composition of the Biosorbent

Quantitative microanalysis of chemical composition was performed using a TESCAN VEGA 3 scanning microscope (TESCAN, Brno, Czech Republic) equipped with an INCA microanalysis system (OXFORD Instruments, High Wycombe, UK). An SE detector (TESCAN, Brno, Czech Republic) was used in the studies. To ensure sample conductivity, dry *Spirulina* sp. mass was placed on a carbon tape of a research table and covered with silver ions in a vacuum sputter. The thickness of the silver layer was about 150 μm.

#### 2.2.2. X-Ray Diffraction Analysis (XRD)

The structural composition of *Spirulina* sp. microalgae was assessed using X-ray diffraction (Bruker D8 Advance with DaVinci, Bruker AXS, Karlsruhe, Germany), with a voltage of 40 kV, Cu X-ray tube 1.5418 Å, diffraction angle (2θ) from 10° to 80°, with 15 rpm rotation. The relative crystallinity was calculated using the following Formula (1):

*Rc* = (*Ac*/(*Ac* + *Aa*)) × 100% (1)

where *Rc* indicates the relative crystallinity of the sample, and *Ac* and *Aa* represent the peak areas of crystalline and amorphous regions, respectively, in the diffractogram. *Ac* and *Aa* were calculated using the Bruker Diffrac.Eva (version 5.2) software.

#### 2.2.3. Fourier Transform Infrared Spectroscopy (FT-IR)

To determine the functional groups responsible for heavy metal biosorption, a Fourier transform infrared (FT-IR) spectrophotometer (Bruker, Billerica, MA, USA) was used in the wavelength range of 400–4000 cm^−1^ with a resolution of 0.7 cm^−1^. FT-IR measurements were performed on samples suspended in KBr discs.

#### 2.2.4. Point of Zero Charge Analysis

The point of zero charge (pH_pzc_) was determined using the pH drift method in a solution of 0.1 M NaCl after initial pH (pH_0_) adjustment in the range of 4–11 with 0.1 M HCl (Sigma-Aldrich, Taufkirchen, Germany) or NaOH (Sigma-Aldrich, Taufkirchen, Germany). After 24 h of contact time, the final pH was measured (pH_f_) by shaking. The pH drift was calculated as the difference between the initial and final pH.

#### 2.2.5. Particle Size and Zeta Potential Analysis

The particle size distribution of *Spirulina* sp. was determined using a laser diffraction analyzer PSA 1190 LD (Anton Paar, Graz, Austria). The measurements were carried out in deionized water after 30 s of ultrasonic dispersion. The zeta potential of the *Spirulina* sp. biomass sample was measured using the electrophoretic light scattering analyzer Litesizer DLS 500 (Anton Paar, Graz, Austria). The measurements were carried out in deionized water at 20 °C and reported as the mean zeta potential.

### 2.3. Metal Ions Speciation Analysis

The speciation of Ni(II) and Pb(II) was performed using Visual MINTEQ 3.0 in the pH range of 1–14. This allowed identification of the dominant metal species and their availability for biosorption.

### 2.4. Batch Adsorption Studies

The adsorption process was studied at different initial concentrations of Ni(II) and Pb(II) ions, 50, 100, 200, 600, 2000, and 5000 mg·L^−1^, respectively. Working solutions were prepared by dissolving analytical salts nickel(II) nitrate hexahydrate (Ni(NO_3_)_2_·6H_2_O) and lead(II) nitrate trihydrate (Pb(NO_3_)_2_) in deionized water. All chemical compounds were from Merck (Sigma-Aldrich, Poznań, Poland). The experiments were carried out with an optimal dose of *Spirulina* sp. of 0.5 g, which was introduced into 50 mL of a solution containing individual metal ions (biosorbent dose of 10 g·L^−1^). The adsorbate and biosorbent mixtures were placed in 100 mL conical flasks and then mixed on a shaker (ELPIN PLUS, type 358A, Mińsk Mazowiecki, Poland) at 120 rpm for 120 min. After the process was completed, the suspensions were decanted and filtered through filter paper. The experiments were carried out under controlled conditions at a temperature of 20 °C and pH = 6. The metal removal efficiency was expressed as the percentage removal of metal ions according to Equation (2). The biosorption capacity (q, mg·g^−1^) of the biosorbent was determined based on Equation (3).

(2) $$\mathrm{Removal}\;(\%)=\frac{\left(C_{0}-C_{e}\right)}{C_{0}}\cdot 100$$

(3) $$\mathrm{Biosorption}\;\mathrm{capacity},\;q\;(\mathrm{mg}\cdot g-1)=\frac{(C_{0}-C_{e})\cdot V_{0}}{m}$$

Here, C_0_ and C_e_ are the initial and final concentrations of Ni(II) and Pb(II) (mg·L^−1^), V is the solution volume (L), and m denotes the mass of the *Spirulina* sp. used (g).

The content of Pb(II) and Ni(II) after the adsorption process was determined on a PERKIN ELMER 3100 (Norwalk, MA, USA) device using the wavelength and slit width appropriate for a given ion, i.e., for Ni(II) 341.5 mm and 0.2 mm and Pb(II) 217 mm and 0.7 mm. Following the device’s work card and the ranges of determination of the analyzed elements, a scale of Ni(II) and Pb(II) ion standards was used with concentrations of 5.0, 10.0, 20.0 mg·L^−1^ and 9.0 and 20.0 mg·L^−1^, respectively.

### 2.5. Equilibrium Isotherm Studies for Adsorption

The adsorption process in the studied system was characterized using selected isothermal models, including the Freundlich, Langmuir, Redlich-Peterson, Jovanović, Halsey, Temkin, Dubinin–Radushevich, and Brunauer-Emmett-Teller (BET) equations, the summary of which is presented in Table 1. To quantitatively assess the fit of individual isothermal models, the coefficients of determination (R^2^) were used. The line graphs present the arithmetic mean obtained from 3 parallel results [*n* = 3].

### 2.6. Adsorption Kinetics Analysis

To determine the kinetics of biosorption of metal ions Ni(II) and Pb(II) by *Spirulina* sp. algae biomass, a series of batch experiments was conducted using a solution with an initial concentration of 600 mg·L^−1^. Six 100 mL conical flasks were filled with 50 mL of metal ion solution and 0.5 g of dry *Spirulina* sp. biomass. Each flask was placed in a shaker and stirred at 120 rpm for the specified times: 15, 30, 60, 90, 120, and 180 min. The experiments were conducted under controlled conditions: temperature 20 °C and pH 6.0. After each contact period, the supernatant was separated from the biomass by filtration and analyzed for the concentration of remaining metal ions.

The mechanism and rate of adsorption of Ni(II) and Pb(II) ions by *Spirulina* sp. biomass were assessed based on fitting experimental data to selected kinetic models. Pseudo-first-order (PFO) (Equation (4)) [72,73] and pseudo-second-order (PSO) (Equation (5)) [74,75] models were analyzed. Additionally, the Weber and Morris intramolecular diffusion model (Equation (6)) [76,77,78] was used, which allows for the identification of the rate-limiting stage of the adsorption process. The obtained data allowed for determining which of the models best describes the course of heavy metal biosorption on the *Spirulina* sp. surface, taking into account the mechanism and rate of the process.

dq_t_/dt = k_1_(q_e_ − q_t_),      ln(q_e_ − q_t_) = −k_1_t + lnq_e_ (4)

dq_t_/dt = k_2_(q_e_ − q_t_)^2^,      t/q_t_ = 1/k_2_q_e_^2^ + t/q_e_ (5)

q_t_ = k’_i_t^1/2^ + b (6)

where q_t_—adsorption capacity (mg·g−1), q_e_—metal ion adsorption capacity at equilibrium (mg·g^−1^), t—adsorption time (min), k_1_—PFO diffusion kinetics adsorption rate constant (min^−1^), k_2_—PSO diffusion kinetics adsorption rate constant (g·mg^−1^·min^−1^), and k_f_—intramolecular diffusion constant.

## 3. Results and Discussion

### 3.1. Characteristics of Spirulina sp.

**Figure 1:**
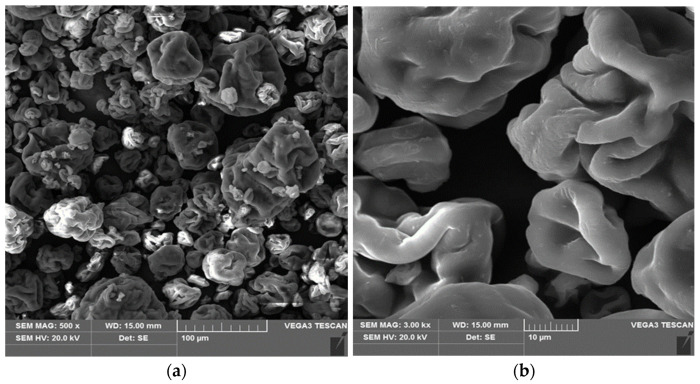
Scanning electron microscopy (SEM) micrographs of the *Spirulina* sp.; magnification: (**a**) 500×; (**b**) 3000×.

The values obtained by scanning electron microscopy (SEM) revealed the surface texture and morphology of *Spirulina* sp. (Figure 1). Algae cells are cylindrical in shape and organized in the form of long, thin threads (often forming clumps or colonies). These threads are covered with a thin cell membrane, and their surface may show various irregularities, such as small depressions or micropores, indicating the presence of surface structures responsible for interactions with the environment and the transport of substances. Analysis of the elemental composition of *Spirulina* sp. showed that the largest percentage share is held by carbon (59.63%) and oxygen (23.32%), which reflects the high content of organic compounds in the biomass structure. Other elements include phosphorus (5.03%), sulfur (3.57%), potassium (5.58%), and calcium (1.83%). The presence of these elements indicates the possibility of participation of functional groups containing P, S, K, and Ca in the mechanisms of metal ion adsorption, which makes *Spirulina* sp. a potentially effective biosorbent in the processes of purifying water contaminated with heavy metals.

**Figure 2:**
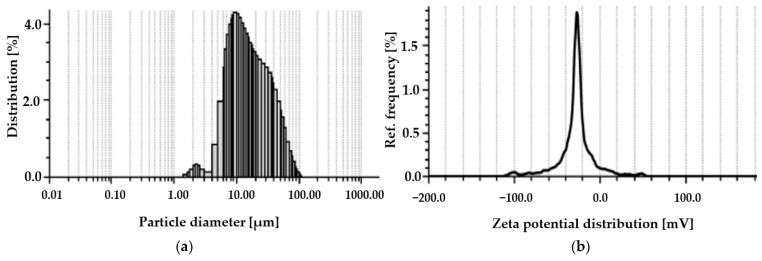
Distribution histogram of *Spirulina* sp.: (**a**) particle diameter; (**b**) zeta potential distribution.

The characteristic sizes D_10_, D_50_, and D_90_ of the tested *Spirulina* sp. are 6.2 μm, 14.5 μm, and 44.1 μm, respectively. The size distribution histogram modeled by volume is shown in Figure 2a. The *Spirulina* sp. sample is composed mainly of micron-sized particles in the 5–50 μm range with the volume weighted median at 14.5 μm, a mean diameter of 21.4 μm, and the majority of particles less than 44.1 μm. The distribution curve is broad and slightly skewed toward larger particles, as indicated by the D_90_ size and the volume span of 2.62. Although the sub-micron particles are not dominant, the material is suitable for applications where surface binding is more critical than high dispersion stability. This size range is consistent with formulations targeting heavy metals biosorption [33,79,80], and can be beneficial due to the ease of the post-treatment separation.

**Figure 3:**
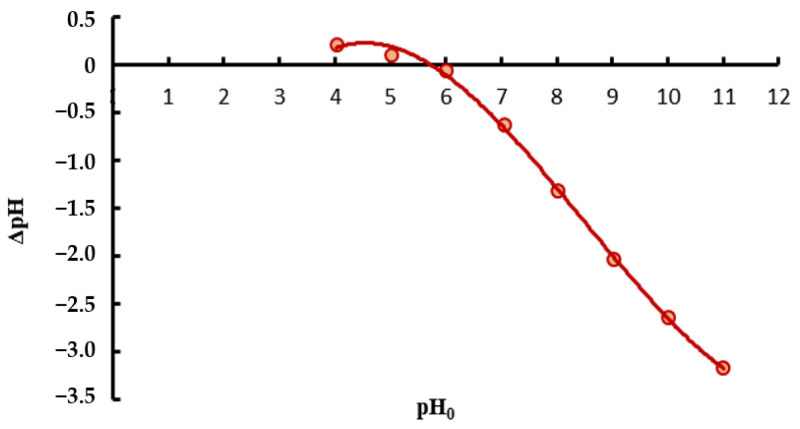
pH point of zero charge of *Spirulina* sp.

The results of zeta potential and pH_pzc_ are shown in Figure 2a and Figure 3, respectively. The average zeta potential at neutral pH of the tested Spirulina is −31.9 mV. That strong negative charge indicates good colloidal stability of dispersion [81] and the affinity for positively charged ions of heavy metals. This relationship is confirmed by the pHpzc value, which is set at a pH of 5.2. This indicates that the surface of spirulina has a slight negative charge in the slightly acidic environment and becomes more negative as the solution becomes more alkaline. Deprotonation of functional groups on the surface of Spirulina increases the binding of metal ions in the pH conditions characteristic of natural waters.

**Figure 4:**
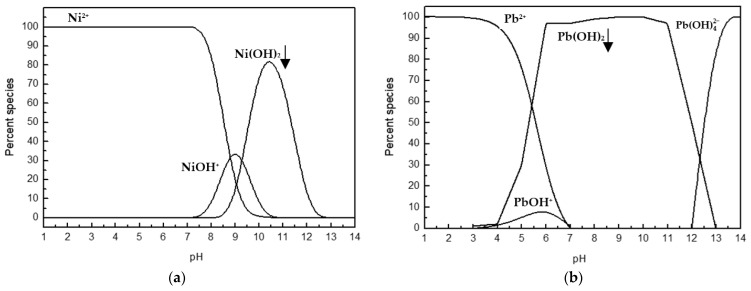
Speciation of (**a**) Ni(II) and (**b**) Pb(II) in aqueous solution as a function of pH.

The speciation of heavy metal ions in aqueous solution plays a crucial role in bio-sorption processes, affecting the availability of ions and the mechanisms by which they bind to the biosorbent surface. For Ni(II) ions, the Ni^2+^ ionic form predominates in solution up to approximately pH 8, meaning that within this pH range, the metal remains soluble and readily available for adsorption (Figure 4). Above this pH, hydroxyl species (NiOH^+^) begin to form, and between pH ~9 and 11, the insoluble Ni(OH)_2_ species dominates. In contrast, Pb(II) ions exhibit a distinctly different speciation pattern—the dominance of Pb^2+^ ends around pH 6, and from pH 7 to 12, the insoluble Pb(OH)_2_ species nearly completely prevails. In highly alkaline conditions (pH > 12), the anionic complex Pb(OH)_4_^2−^ is also present, which does not occur with nickel. These differences likely stem from the distinct chemical properties of the metals, such as ionic radius, hydration energy, and affinity for functional groups present on the biomass surface. Consequently, the optimal pH range for Pb(II) biosorption is shifted toward lower values compared to Ni(II), which should be taken into account when designing effective processes for heavy metal removal from water and wastewater using *Spirulina* sp. biomass.

**Figure 5:**
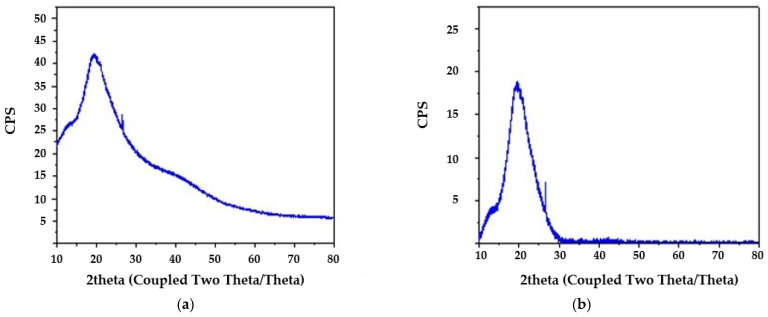
XRD patterns of *Spirulina* sp.: (**a**) original spectrum; (**b**) baseline-corrected spectrum.

*Spirulina* sp. shows mainly an amorphous structure, which means that there is no clear, ordered arrangement of molecules characteristic of crystalline materials (Figure 5). Nevertheless, an intense diffraction peak was observed at an angle of about 19.4° (2θ), which may indicate the presence of local areas of partial structural order. The calculated relative crystallinity of *Spirulina* sp. was 44.7%, which suggests that almost half of its structure shows some features of crystalline order, while the remaining part remains in the amorphous state.

**Figure 6:**
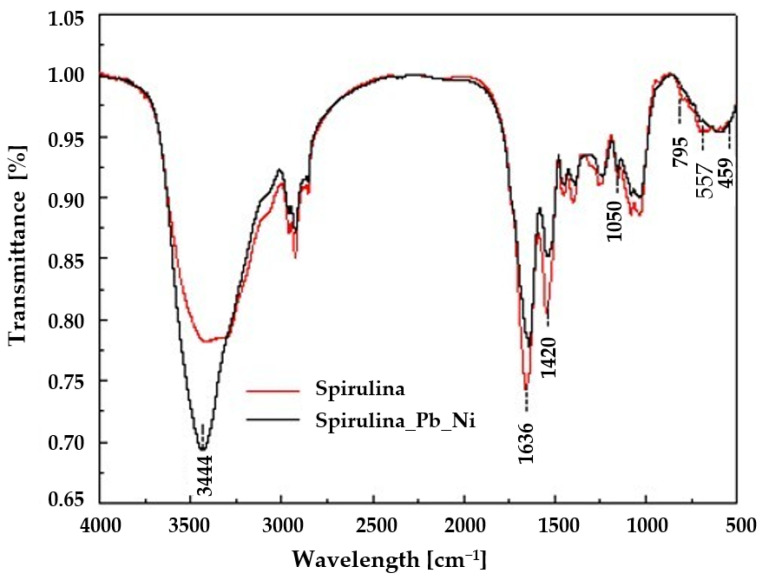
FT-IR spectra of the *Spirulina* sp.

FTIR spectra of pure *Spirulina* sp. and after adsorption of Ni(II) and Pb(II) ions are shown in Figure 6. Numerous characteristic bands are visible, indicating the presence of active functional groups involved in the adsorption process. The broad band observed around ~3400 cm^−1^ is attributed to the stretching vibrations of hydroxyl (–OH) and amine (–NH) groups, which are typical for proteins and polysaccharides present in Spirulina sp. After heavy metal adsorption, this band showed a slight shift and decreased intensity, suggesting the involvement of these groups in hydrogen bonding and metal ion complexation. The bands in the range of 1640–1540 cm^−1^ correspond to amide I and II bands, related to the C=O and N–H vibrations of amide groups, respectively. Their shifts toward lower wavenumbers after adsorption indicate a direct interaction between protein amide groups and Ni(II) and Pb(II) ions, suggesting the formation of coordination complexes between oxygen or nitrogen atoms and metal cations. Changes were also observed in the 1400–1000 cm^−1^ region, where bands attributed to the stretching vibrations of C–O, C–N, –COOH, and PO_4_^3−^ groups occur. After metal adsorption, these bands were significantly weakened and shifted, indicating the involvement of carboxyl and phosphate groups in the adsorption process. In the case of carboxyl groups, this mechanism may involve both complexation of metal cations with the lone electron pairs of oxygen atoms and ion exchange with the protons of –COOH groups. Similarly, phosphate groups can participate in metal coordination via oxygen atoms, leading to the formation of stable complexes. Additionally, in the 900–600 cm^−1^ region, changes in the shape and intensity of the bands were observed, confirming the modification of the *Spirulina* sp. surface structure upon contact with metal ions. Based on the FTIR band shift analysis and pH_pzc_ results, it can be concluded that the adsorption process of Ni(II) and Pb(II) primarily occurs through chemisorption mechanisms such as complexation or chelation by hydroxyl, carboxyl, amine, and phosphate groups, with a possible additional contribution of ion exchange in the case of carboxyl and phosphate groups. The presence of these functional groups provides numerous binding sites for metal ions, enhancing the adsorption efficiency of *Spirulina* sp.

### 3.2. Batch Adsorption Experiments

**Figure 7:**
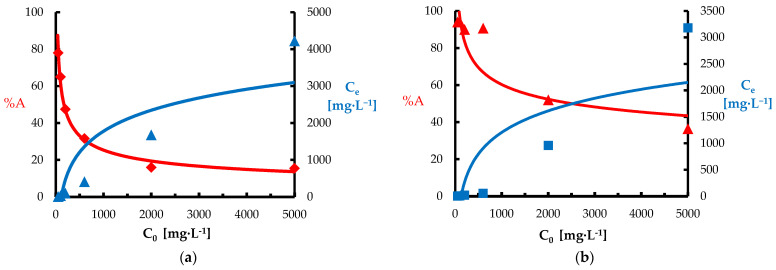
Dependence of adsorption efficiency on initial ion concentration: (**a**) Ni(II); (**b**) Pb(II) (biosorbent dose 10 g·L^−1^, T = 20 °C, C_0_ = 50–5000 mg·L^−1^, pH = 6, mixing time 120 min).

Figure 7 shows the effect of the initial concentration of Ni(II) and Pb(II) ions on the efficiency of their removal from the aqueous solution. In the case of both metals, a classic trend characteristic of biosorption was observed: the percentage removal (%A) decreases with the increase of the initial C_0_ concentration. For Ni(II), the maximum adsorption efficiency at low C_0_ values exceeded 80%, but at the highest concentration (5000 mg·L^−1^), it decreased to about 15%. The C_e_ values increased exponentially, which suggests a rapid saturation of the *Spirulina* sp. surface. For Pb(II), a higher removal efficiency was observed, which in the low concentration range reached even 95%. With the increase of C_0_, the %A value gradually decreased, but still remained at a higher level than for Ni(II), which indicates a stronger binding of lead by the *Spirulina* sp. surface. The adsorption order was as follows: Ni(II) < Pb(II). In this case, Pb(II) ions, despite having a larger ionic radius (1.2 Å) than Ni(II) (0.8 Å), showed stronger interactions with functional groups present on the surface of Spirulina sp. biomass. However, it should be noted that the studies were performed at pH = 6, which could result in the formation of a small amount of Pb(OH)_2_. High efficiency of heavy metal removal by *Spirulina* sp. at low concentrations indicates its potential as a cheap and ecological biosorbent. However, at high ion concentrations, it may be necessary to use a larger amount of biomass or stepwise adsorption to obtain the desired purification efficiency.

### 3.3. Equilibrium Adsorption

**Figure 8:**
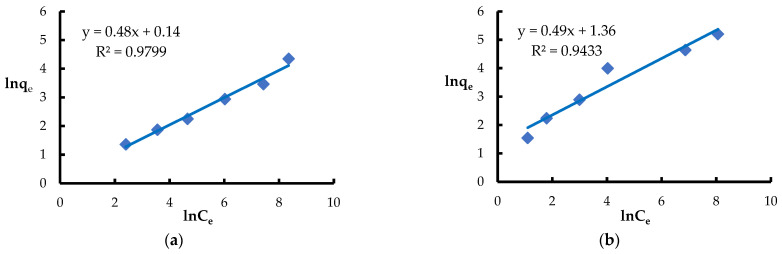
Fitting the Freundlich model to the adsorption of (**a**) Ni(II) ions; (**b**) Pb(II) ions (biosorbent dose 10 g·L^−1^, T = 20 °C, C_0_ = 50–5000 mg·L^−1^, pH = 6, mixing time 120 min).

Based on the obtained results, it can be stated that the process of adsorption of heavy metal ions by *Spirulina* sp. biomass is dynamic and strongly dependent on the initial concentration of metals in the solution. In order to better understand the adsorption mechanism and determine the process rate and the stages limiting its rate, an analysis of adsorption kinetics was carried out using selected mathematical models. Among the models used, the Freundlich isotherm—an empirical equation describing adsorption on heterogeneous surfaces—showed a good fit to the experimental data [66,82] (Figure 8). The values of the 1/n parameter obtained for Ni(II) and Pb(II) ions were 0.48 and 0.49, respectively, which correspond to n values of 2.1 and 2.0 (Table 2). These parameters indicate a favorable course of the adsorption process and energetic differentiation of active sites on the biosorbent surface. High values of the coefficient of determination (R^2^), amounting to 0.98 for Ni(II) and 0.94 for Pb(II), confirm the good fit of the data to the Freundlich model and thus its suitability for describing the occurring adsorption processes.

**Figure 9:**
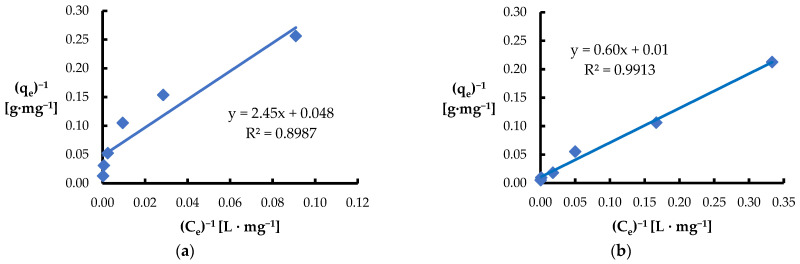
Fitting the Langmuir model to the adsorption of (**a**) Ni(II) ions; (**b**) Pb(II) ions (biosorbent dose 10 g·L^−1^, T = 20 °C, C_0_ = 50–5000 mg·L^−1^, pH = 6, mixing time 120 min).

The second model used was the Langmuir isotherm, which assumes that adsorption occurs on a homogeneous surface by forming a monolayer, and the number of available adsorption sites is limited (Figure 9) [65,82,83]. The maximum adsorption capacity of *Spirulina* sp. at pH 7 was 20.8 mg·g^−1^ for Ni(II) and 93.5 mg·g^−1^ for Pb(II) (Table 2). Analysis of these results suggests that the surface of *Spirulina* sp. has a higher affinity for lead ions than for nickel ions. The observed adsorption selectivity may be related to differences in the ionic radii of ions in the hydrated state—Pb(II) has a smaller radius (4.01 Å) compared to Ni(II) (4.04 Å). A smaller number of water molecules surrounding lead ions may facilitate their migration to the adsorbent surface, which translates into their higher adsorption efficiency (adsorption sequence: Pb(II) > Ni(II)).

**Figure 10:**
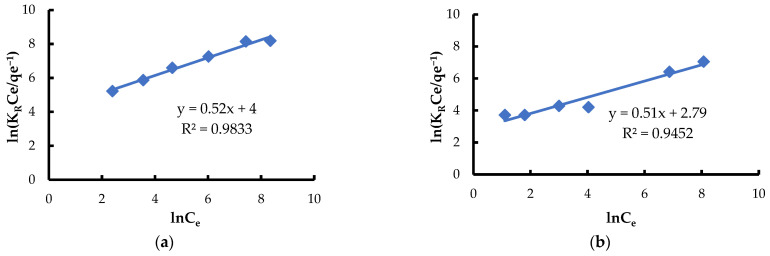
Fitting the Redlich-Peterson model to the adsorption of (**a**) Ni(II) ions; (**b**) Pb(II) ions (biosorbent dose 10 g·L^−1^, T = 20 °C, C_0_ = 50–5000 mg·L^−1^, pH = 6, mixing time 120 min).

In turn, the Redlich-Peterson isotherm, which is a three-parameter model, is applicable to both homogeneous and heterogeneous systems [84,85,86]. Analysis of experimental data has shown that this model describes well the adsorption process of the tested ions on *Spirulina* sp., as evidenced by the high value of the determination coefficient (R^2^ = 0.9452 for Pb(II) and R^2^ = 0.9833 for Ni(II)) (Figure 10). The parameter β, which takes values from 0 to 1, reflects the nature of adsorption. When β approaches 1, the model resembles the Langmuir isotherm, which suggests a homogeneous adsorbent surface and monolayer adsorption. When β approaches 0, the model resembles the Freundlich isotherm, indicating the heterogeneity of the adsorbent surface and multilayer adsorption. In the analyzed case, the β value was 0.51 and 0.52 for Pb(II) and Ni(II) (Table 2), which suggests that the surface of *Spirulina* sp. has diverse active sites and the adsorption process is of a mixed nature—it occurs partly on homogeneous and partly on heterogeneous adsorption sites. Such values also indicate a non-ideal monolayer—there may be effects of interactions between adsorbed ions (Pb(II) and Ni(II)), as well as the possibility of multilayer adsorption to a certain extent.

**Figure 11:**
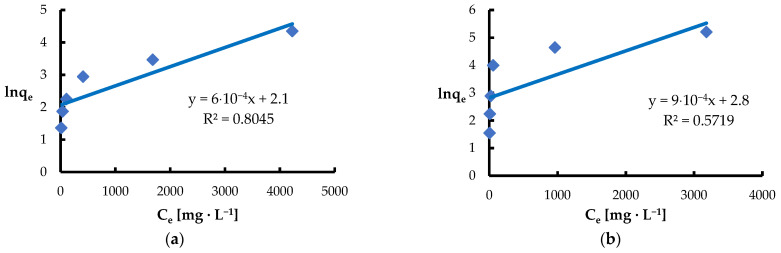
Fitting the Jovanović model to the adsorption of (**a**) Ni(II) ions; (**b**) Pb(II) ions (biosorbent dose 10 g·L^−1^, T = 20 °C, C_0_ = 50–5000 mg·L^−1^, pH = 6, mixing time 120 min).

The adsorption model developed by Jovanović [70] is based on assumptions similar to those used in the Langmuir isotherm, assuming the formation of a monolayer on the adsorbent surface (Figure 11). In contrast to the classical Langmuir model, the Jovanović isotherm takes into account some energetic differentiation of adsorption sites and their heterogeneous nature. According to the obtained results, the maximum adsorption capacity (q_max_) for *Spirulina* sp. was 8.2 mg·g^−1^ for Ni(II) and 16.4 mg·g^−1^ for Pb(II) (Table 2), which indicates a significantly greater ability of the biosorbent to bind lead ions.

**Figure 12:**
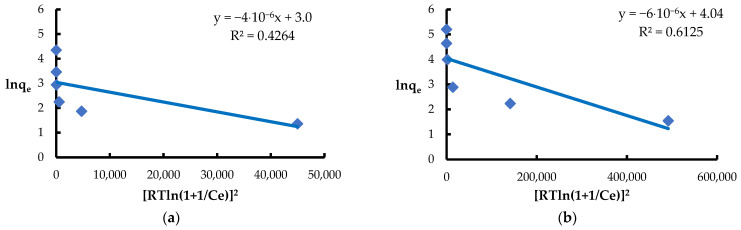
Fitting the Dubinin–Radushkevich model to the adsorption of (**a**) Ni(II) ions; (**b**) Pb(II) ions (biosorbent dose 10 g·L^−1^, T = 20 °C, C_0_ = 50–5000 mg·L^−1^, pH = 6, mixing time 120 min).

The Dubinin–Radushkevich isotherm is used to analyze the adsorption mechanism on heterogeneous surfaces [87,88]. This model shows good agreement with experimental data at higher metal concentrations, but its validity decreases at low ion concentrations. An important parameter of this isotherm is the mean free energy of adsorption (E), which allows for assessing whether the dominant mechanism is physisorption or chemisorption [89]. Since the Dubinin–Radushkevich model takes into account the effect of temperature, the data are presented as the dependence of the logarithm of the amount of adsorbed substance on the square of the potential energy [87]. In the analyzed case of Pb(II) and Ni(II) adsorption, the obtained E values were 0.29 kJ·mol^−1^ and 0.35 kJ·mol^−1^, respectively, which indicates the dominance of physical processes in the ion binding mechanism [73,82]. For comparison, the energy range characteristic for the ion exchange mechanism is in the range of 8–16 kJ·mol^−1^ [73]. In turn, the maximum amounts of adsorbed ions in the equilibrium state were 56.8 mg·g^−1^ for Pb(II) (R^2^ = 0.6125) and 20.09 mg·g^−1^ for Ni(II) (R^2^ = 0.4264), which indicates a moderate fit of the Dubinin–Radushkevich model to the obtained experimental data (Figure 12, Table 2).

**Figure 13:**
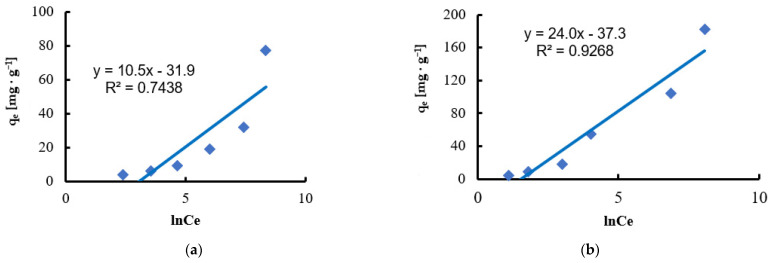
Fitting the Temkin model to the adsorption of (**a**) Ni(II) ions; (**b**) Pb(II) ions (biosorbent dose 10 g·L^−1^, T = 20 °C, C_0_ = 50–5000 mg·L^−1^, pH = 6, mixing time 120 min).

The Temkin model [90] takes into account the interactions between the adsorbent surface and the adsorbate molecules, assuming that the heat of adsorption decreases linearly with increasing surface coverage [87]. Although the Temkin isotherm was originally developed to describe adsorption in gaseous systems, its application to liquid systems is limited and does not always provide a good fit [91]. Based on the linear interpretation of the data (Figure 13), the free energy of adsorption values of 232 J·mol^−1^ for Ni(II) and 101 J·mol^−1^ for Pb(II) were determined, suggesting the dominance of physisorption as the adsorption mechanism. The coefficients of determination R^2^ obtained for this model were 0.7438 for Ni(II) and 0.9268 for Pb(II), respectively, which indicates a better fit than the Dubinin–Radushkevich isotherm.

**Figure 14:**
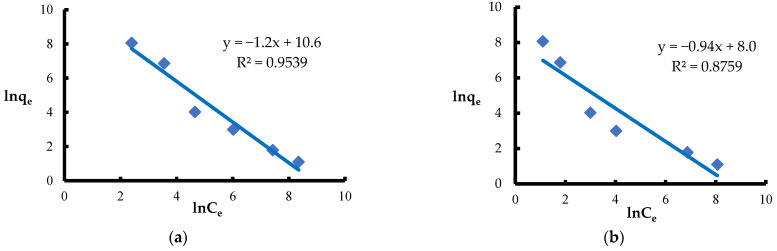
Fitting the Halsey model to the adsorption of (**a**) Ni(II) ions; (**b**) Pb(II) ions (biosorbent dose 10 g·L^−1^, T = 20 °C, C_0_ = 50–5000 mg·L^−1^, pH = 6, mixing time 120 min).

**Figure 15:**
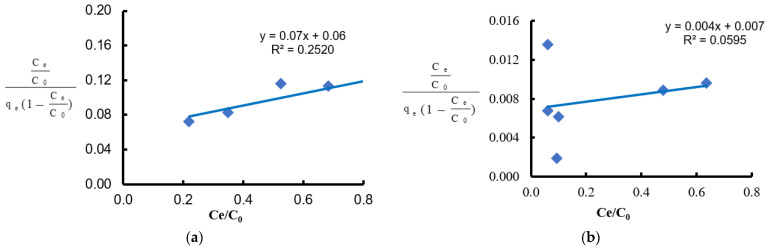
Fitting the BET model to the adsorption of (**a**) Ni(II) ions; (**b**) Pb(II) ions (biosorbent dose 10 g·L^−1^, T = 20 °C, C_0_ = 50–5000 mg·L^−1^, pH = 6, mixing time 120 min).

The Halsey isotherm [71] is used to describe multilayer adsorption on heterogeneous surfaces, assuming a decrease in adsorption energy with the layer thickness. This model, similarly to the BET isotherm [92], works well at high concentrations and pressures, especially in liquid–solid systems [87]. Both models are based on the assumption of an exponential increase in the number of adsorption sites with advancing adsorption, which suggests a mechanism for the formation of multilayers. However, the values of the coefficient of determination R^2^ (Table 2) obtained for the Halsey and BET models were noticeably lower compared to the models describing monolayer adsorption. This indicates that the multilayer adsorption concept was not suitable for describing the binding process of Pb(II) and Ni(II) ions on the surface of *Spirulina* sp. under the conditions studied (Figure 14 and Figure 15).

Comparative analysis of the obtained R^2^ determination coefficients showed that the Langmuir, Jovanović, and Redlich-Peterson isotherms best represented the adsorption process of Ni(II) and Pb(II) ions on *Spirulina* sp. (Table 2). These models were characterized by the highest degree of fit to the experimental data, which is confirmed by high R^2^ values. Based on the analysis of the fitting errors of individual models for both analyzed ions, the following order of accuracy of the adsorption process representation was established: Freundlich > Redlich-Peterson > Langmuir > Halsey > Jovanović > Temkin > Dubinin–Radushkevich > BET. Additionally, the estimated maximum adsorption capacity of the monolayer based on the Langmuir model, which was 20.8 mg·g^−1^ for Ni(II) and 93.5 mg·g^−1^ for Pb(II), respectively, demonstrates the favorable adsorption properties of *Spirulina* sp., which makes it a promising and economical material for the removal of heavy metal ions from aqueous solutions.

### 3.4. Kinetic Adsorption

**Figure 16:**
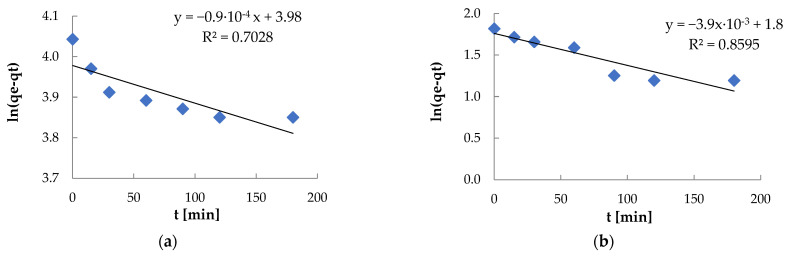
Kinetic curve of the pseudo-first-order model: (**a**) for Ni(II) ions; (**b**) for Pb(II) ions (biosorbent dose 10 g·L^−1^, T = 20 °C, C_0_ = 600 mg·L^−1^, pH = 6, contact time 15–180 min).

**Figure 17:**
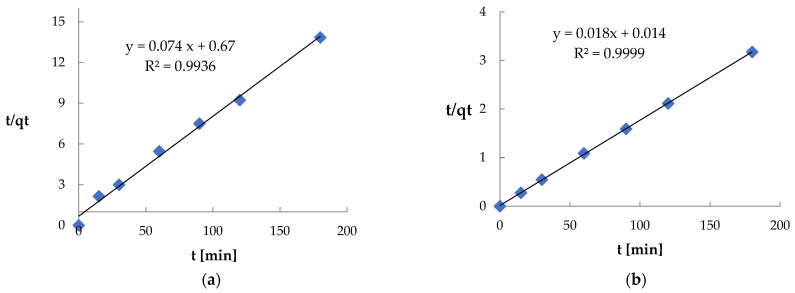
Kinetic curve of the pseudo-second-order model: (**a**) for Ni(II) ions; (**b**) for Pb(II) ions (biosorbent dose 10 g·L^−1^, T = 20 °C, C_0_ = 600 mg·L^−1^, pH = 6, contact time 15–180 min).

The mechanism and rate of adsorption of Ni(II) and Pb(II) ions by Spirulina sp. were assessed based on pseudo-first-order (Figure 16) and pseudo-second-order (Figure 17) kinetic models. The pseudo-first-order model did not reflect the course of the process (kinetic curves did not form a straight line, and lower R^2^ values were obtained: 0.7028 for Ni(II), 0.8595 for Pb(II)). However, the data were well fitted to the pseudo-second-order model, which is confirmed by high values of determination coefficients (R^2^ = 0.9936 and 0.9999), indicating the dominance of chemisorption [74].

**Figure 18:**
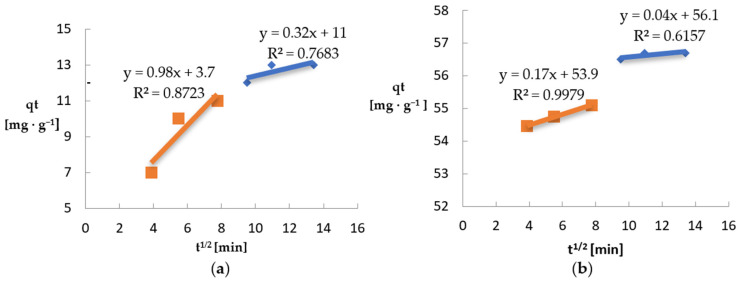
Model of intra-grain diffusion: (**a**) Ni(II) ions; (**b**) Pb(II) ions (biosorbent dose 10 g·L^−1^, T = 20 °C, C_0_ = 600 mg·L^−1^, pH = 6, contact time 15–180 min).

The multilinear plots of qt = k’it1/2 + bi indicate two stages of adsorption of Ni(II) and Pb(II) ions [93] (Figure 18, Table 3). The first stage is characterized by a linear relationship with a high coefficient of determination (R^2^ = 0.8723 and 0.9979), which suggests that in the initial stage of adsorption, the dominant mechanism is the diffusion of metal ions to the biomass surface. The second stage (blue line) has a lower value of R^2^ (0.7683 and 0.6157), which may indicate a more complex mechanism of intrapore diffusion or the influence of other factors, such as limited availability of free adsorption sites or effects related to adsorption equilibrium. The variation of the line slope and the decrease of the R^2^ value in the second stage confirm that internal diffusion is not the only factor limiting the adsorption rate of Ni(II) and Pb(II)—the influence of the boundary layer and the biosorbent structure may also be significant. The values of the micropore diffusion constant k’2 for Ni(II) (0.32) and Pb(II) (0.04) were lower than the values of the macropore diffusion constants k’1 (0.98 and 0.17 for Ni(II) and Pb(II)).

**Figure 19:**
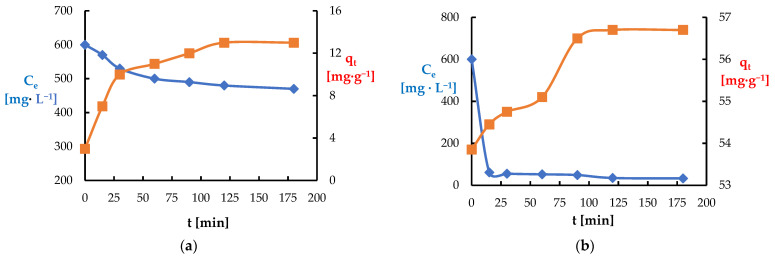
Dependence of mixing time on the adsorption of metal ions: (**a**) Ni(II); (**b**) Pb(II) (biosorbent dose 10 g·L^−1^, T = 20 °C, C_0_ = 600 mg·L^−1^, pH = 6, contact time 15–180 min).

To assess the effect of contact time on the adsorption of Ni(II) and Pb(II) ions on *Spirulina* sp., the changes in the concentration of ions in the solution and the amount of adsorbed substance were analyzed as a function of time (Figure 19). In both cases, an exponential increase in adsorption was observed with a simultaneous decrease in the concentration of metals in the solution, with the fastest course in the first minutes until equilibrium was reached. The adsorption of Pb(II) ions is faster and more efficient than that of Ni(II), and the equilibrium state for Pb(II) was reached after about 40 min. The adsorption rate of Ni(II) was initially faster than that of Pb(II).

*Spirulina* sp. shows high potential as an effective biosorbent for the removal of heavy metal ions such as lead and nickel. Numerous scientific studies have also confirmed its adsorption capacity and practical application in water environment purification processes. The results of these studies are summarized in Table 4, which presents the wide range of applications and effectiveness of *Spirulina* sp. as a natural and environmentally friendly sorbent. However, it should be noted that the efficiency of this biosorbent largely depends on the precise adjustment of process conditions to the characteristics of specific pollutants, highlighting the need to optimize adsorption parameters to achieve the best results in practical applications. To emphasize the competitiveness of *Spirulina sp*. as a biosorbent, its adsorption capacity and efficiency in removing Ni(II) and Pb(II) were compared with other biosorbents (Table 5). In this study, *Spirulina* sp. exhibited an adsorption capacity of approximately 20.8 mg·g^−1^ for Ni(II) and 93.5 mg·g^−1^ for Pb(II), which is comparable to or higher than biomaterials such as *Cannabis sativa* L. (17.1 mg·g^−1^ for Ni and 15.4 mg·g^−1^ for Pb) [94], milkweed fibers (3.67 mg·g^−1^ for Ni and 18.79 mg·g^−1^ for Pb) [95], or cocoa pod husk biomass (14.3 mg·g^−1^ for Ni and 25.2 mg·g^−1^ for Pb) [96]. Importantly, *Spirulina sp.* also demonstrates competitive potential compared to more advanced materials, such as functionalized nanoadsorbents diethylene triamine penta (methylene phosphonic acid) (8.56 mg·g^−1^ for Ni and 13.28 mg·g^−1^ for Pb) [97] and semicarbazide modified poly (methylmethacrylate) (11.53 mg·g^−1^ for Ni and 32.36 mg·g^−1^ for Pb) [98]. It is worth emphasizing that, unlike many previous studies focusing on low concentrations of heavy metals, the present study applied relatively high concentrations of Pb(II) and Ni(II). This approach allowed for a detailed assessment of the adsorption capacity limits of Spirulina sp. and provided insights into the adsorption mechanisms under conditions of intense contaminant loading, which yields valuable information about its potential for application in heavily polluted aquatic environments.

It should also be noted that *Spirulina* sp. can be subjected to regeneration processes, which play a key role in assessing its economic feasibility [99,100]. Regeneration can be carried out using various methods, including diluted mineral acids (e.g., HCl, HNO_3_), EDTA, and salt solutions (NaCl). Diluted acids can protonate functional groups on the biomass surface, leading to desorption of metal ions via ion exchange and breakdown of surface complexes. Studies have shown that using 0.1 M HCl enables the recovery of more than 84% of Pb(II) ions adsorbed onto the biosorbent surface [99]. On the other hand, complexing agents such as EDTA form strong, soluble complexes with metal ions, facilitating their removal from the adsorbent surface, although economic and environmental aspects of their application should be considered. Salt solutions with high ionic strength can induce ion exchange, but their efficiency is generally lower compared to acid or chelating methods.

It is worth emphasizing that *Spirulina* sp. biomass can be produced in a controlled manner on an industrial scale, which further increases its attractiveness as a biosorbent for heavy metals. Nevertheless, the regeneration efficiency and stability of *Spirulina* sp. during multiple adsorption–desorption cycles require experimental confirmation to assess potential structural changes or reductions in adsorption capacity. Therefore, *Spirulina* sp. constitutes a competitive, efficient, and sustainable biosorbent for heavy metals with high application potential.

**Table 4:** Overview of adsorption studies using spirulina-based materials for heavy metals.

Heavy Metals	Adsorption Capacity	Experimental Conditions	
Ni(II) Pb(II)	20.8 mg·g^−1^ 93.5 mg·g^−1^,	*Spirulina* sp. dry biomass; pH: 6 and 20 °C; Contact time: 120 min; Biomass dosage: 10 g·L^−1^; Initial concentration: 50–5000 mg·L^−1^	[this study]
Cd	98 mg·g^−1^	*Spirulina (Arthrospira)* platensis TISTR 8217 dry biomass; pH: 7 and 26 °C; Contact time: 30 min (equilibrium reached); Biomass dosage: 0.5 g·L^−1^	[35]
Cr(III)	185 mg·g^−1^	*Spirulina* sp. (*Lyophilized form–L* and *Photoautotrophic form–A*); pH: 7 and 35 °C; Contact time: 30 min (kinetic equilibrium reached within ca. 10 min); Biomass dosage: 1 g·L^−1^	[36]
Cd(II)	159 mg·g^−1^		
Cu(II)	196 mg·g^−1^		
Cd(II)	625 mg·g^−1^	Live *Spirulina* sp. and Dead *Spirulina* sp. (sun dried); pH: 6.0 and 35–38 °C; Contact time: About 960 min (for final reading, kinetics measured up to 240 min); Biomass dosage: 12 g·L^−1^	[61]
Cd(II)	355 mg·g^−1^		
Cr(VI)	189 mg·g^−1^	Fresh *Spirulina platensis* (dried at 60 °C) and Spent *Spirulina platensis* biomass (after b-carotene extraction with acetone, dried at 60 °C); pH: 1.5 and 25 °C; Contact time: kinetics studied up to 600 min; Biomass dosage: 1.0 g·L^−1^	[37]
Cr(VI)	213 mg·g^−1^		
Cr(VI)	99% removal	*Spirulina platensis* nanoparticles (prepared by mechanical agitation at 10,000 rpm, 20 min; mean diameter 215.6 nm, PDI 0.151); pH: 4 and 25 °C; Contact time: Agitated until equilibrium; Initial concentration: 250 mg· L^−1^; Biomass dosage: 1.0 g·L^−1^	[38]
Pb(II)	253 mg·g^−1^	Vacuum freeze-dried *Spirulina platensis* (Vsp), prepared at <10 Pa and ~60 °C for 12 h after pre-freezing at ~80 °C; pH: 5.0 and 25 °C; Contact time: 720 min; Initial concentration: from 0 to 100 mg·L^−1^; Biomass dosage: 0.5 g·L^−1^	[39]
As(V)	25 mg·g^−1^	*Spirulina platensis* modified with 0.5 mol/L ZnCl_2_ for 24 h at 25 °C, dried at 80 °C, sieved (<500 µm); Research conducted in the binary system: As (V) and Cd(II); pH: 6.0 and 25 °C; Contact time: 480 min; Initial concentration: from 10 to 300 mg·L^−1^; Biomass dosage: 4 g·L^−1^	[40]
Cd(II)	29 mg·g^−1^		
Zn(II)	51 mg·g^−1^	*Arthrospira (Spirulina) platensis* biomass dried at 60 °C and ground into powder; pH: 6 and 25 °C; Contact time: 60 min; Initial concentration: from 20 to 100 mg·L^−1^; Biomass dosage: 3 g·L^−1^	[41]
Cr(VI)	4 mg·g^−1^	*Spirulina platensis* modified with 5% Na_2_CO_3_, 5% KCl, and 5% Na_2_CO_3_, dried at 80 °C, grounded, sieved (<10 mm); Research conducted in the binary system: Cr(VI) and Cu(II), Fe(II) and Cr(VI), Cu (II) and Cr (VI), Ni (II) and Cr (VI); Initial concentration: from 25 to 150 mg·L^−1^; pH: 4 and 25 °C; Biomass dosage: 6 g·L^−1^; Contact time: 120 min	[42]
Fe(II)	18 mg·g^−1^		
Cu(II)	6 mg·g^−1^		
Ni(II)	12 mg·g^−1^		
Pb(II)	88 mg·g^−1^	*Spirulina platensis* immobilized in alginate beads (S.P@Ca-SA), prepared with 2% S. platensis, 2% SA, 4% CaCl_2_; pH: 5 and 25 °C; Contact time: Equilibrium time, within 6 h; Biomass dosage: 1, 5, and 10 g·L^−1^	[101]
Pb(II)	115 mg·g^−1^	*Spirulina* (Free biomass); pH 5.2 and 25 °C; Contact time: 72 h; Initial concentration: 0.6 to 5.6 mg· L^−1^; Biomass dosage: 50 mg·L^−1^	[102]
Pb(II)	282 mg·g^−1^	*Spirulina* (Immobilized on alginate); pH: 5.2 and 25 °C; Contact time: Within 360 min (in fixed-bed column); Initial concentration: Varied for isotherm (20 to 150 mg·L^−1^), varied for column (4 to 20 mg·L^−1^)	
Pb	0.6 mg/10^5^ alga cells	*Spirulina* (Live biomass); Contact time: Equilibrium within 1440 min (rapid adsorption in first 0–12 min); Temperature: 25 °C; Initial concentration: from 10 to 50 mg·L^−1^	[103]
Zr(IV)	110 mg·g^−1^	*Spirulina platensis* (Dry biomass); pH 2 and 25 °C; Contact time: 35 min; Initial concentration: 150 mg·L^−1^; Biomass dosage: 2.5 g·L^−1^	[104]
Cr(III) Mn(II) Mg(II)	45 mg·g^−1^ 44 mg·g^−1^ 42 mg·g^−1^	Raw *Arthrospira (Spirulina) platensis* biomass; pH: 5; Biomass dosage: 1 g· L^−1^; Equilibrium time: ~50 min; Equilibrium conditions tested for concentrations 10–300 mg·L^−1^	[105]

**Table 5:** Comparison of various biosorbents used for the removal of Ni(II) and Pb(II) ions from aqueous solutions.

Material	Adsorption Capacity	Experimental Conditions	
SYNTHETIC ADSORBENTS			
Diethylene triamine penta (methylene phosphonic acid) functionalized magnetic nanoadsorbent Fe_3_O_4_@SiO_2_-DTPMP	13.28 mg·g^−1^ (Pb) 8.56 mg·g^−1^ (Ni)	For Pb at pH 6, for Ni at pH 5; Temperature 25 °C; Biomass dosage: 1 g·L^−1^; Initial concentration: 10–50 mg·L^−1^	[97]
Semicarbazide modified poly(methylmethacrylate)	32.36 mg·g^−1^ (Pb) 11.53 mg·g^−1^ (Ni)	pH 5.0; Temperature 25 °C; Biomass dosage: 1,5 g·L^−1^ (Pb), 1.0 g·L^−1^ (Ni); Initial concentration: 10–60 mg·L^−1^	[98]
BIOADSORBENTS			
*Spirulina* platensis dry biomass	20.8 mg·g^−1^ (Ni) 93.5 mg·g^−1^ (Pb)	pH: 6; Temperature 20 °C; Contact time: 120 min Biomass dosage: 10 g·L^−1^; Initial concentration: 50–5000 mg·L^−1^	[This study]
Bioadsorbents composed of *Cannabis sativa* L. leaves extract and fibers	15.384 mg·g^−1^ (Pb) 17.137 mg·g^−1^ (Ni)	Temperature 25 °C; Biomass dosage: 0.33–0.25 g·L^−1^; Initial concentration: 4–8 mg·L^−1^	[94]
Sugarcane bagasse	44.053 mg·g^−1^ (Pb) 56.818 mg·g^−1^ (Ni)	pH 5.0; Temperature 25 °C; Biomass dosage: 0.1 g·L^−1^; Initial concentration: 2–60 mg·L^−1^; Contact time: 60 min	[106]
Sargassum filipendula-brown alga	367.9 mg·g^−1^ (Pb) 34.3 mg·g^−1^ (Ni)	pH 5.0; Temperature 25 °C; Biomass dosage: 2 g·L^−1^; Initial concentration: 50–150 mg·L^−1^; Contact time: 85 min	[107]
Milkweed fibers	18.79 mg·g^−1^ (Pb) 3.67 mg·g^−1^ (Ni)	pH 6.0; Temperature 25 °C; Biomass dosage: 2 g·L^−1^; Initial concentration: 5–80 mg·L^−1^	[95]
Biochar produced from Eucalyptus camdulensis sawdust	193.95 mg·g^−1^ (Pb) 55.21 mg·g^−1^ (Ni)	pH 6.0; Temperature 25 °C; Biomass dosage: 0.4 g·L^−1^ (Pb) and 0.2 g·L^−1^ (Ni); Initial concentration: 20 mg·L^−1^ (Pb) and 40 mg·L^−1^ (Ni)	[108]
Cocoa (*Theobroma cacao* L.) pod husks	25.2 mg·g^−1^ (Pb) 14.31 mg·g^−1^ (Ni)	pH 6.0; Temperature 25 °C; Initial concentration: 100 mg·L^−1^ (Pb) and 40 mg·L^−1^ (Ni); Fixed-Bed Column Study (Bed depth: 4 and 7.5 cm; diameter of 6.6 cm; flow rate of 1 mL/s)	[96]
MINERAL ADSORBENTS			
Alkali-activated Egyptian calcium bentonite	13.0 mg·g^−1^ (Pb) 12.2 mg·g^−1^ (Ni)	pH 7.0; Temperature 20 °C; Biomass dosage: 1 g·L^−1^; Initial concentration: 10–50 mg·L^−1^; Contact time: 120 min	[109]
Manganese oxides (α-MnO_2_, β-MnO_2_, γ-MnO_2_, δ-MnO_2_, λ-MnO_2_)	40.3 ÷299.2 mg·g^−1^ (Pb)	pH 4.0; room temperature; Biomass dosage: 0.5 g·L^−1^; Initial concentration: 45 mg·L^−1^ for α-, β-, γ-, and λ-MnO_2_, and 150 mg·L^−1^ for δ-MnO_2_	[110]

## 4. Conclusions

*Spirulina* sp. shows high potential as an effective and ecological biosorbent for removing Ni(II) and Pb(II) ions from aqueous solutions, constituting an economical alternative to traditional adsorbents such as activated carbon.

The cellular structure of *Spirulina* sp. and the presence of active functional groups (hydroxyl, amino, carboxyl, phosphate) promote effective adsorption of heavy metal ions through complexation mechanisms and coordination bonds. The dominant elements in the composition of *Spirulina* sp. are carbon (59.63%) and oxygen (23.32%), with the presence of phosphorus, sulfur, potassium, and calcium. The material is characterized by a favorable particle size (average 14.5 μm) and negative zeta potential (−31.9 mV at neutral pH), which ensures colloidal stability and strong affinity for positively charged metal ions.

The adsorption of Pb(II) ions is more effective than that of Ni(II), reaching up to 95% removal at low concentrations, which results, among others, from differences in ionic radius and adsorption properties. The Langmuir, Jovanović, and Redlich-Peterson isotherm models describe the adsorption process well, confirming the maximum adsorption capacities at the level of 93.5 mg·g^−1^ for Pb(II) and 20.8 mg·g^−1^ for Ni(II). Low values of the average free energy of adsorption (E) in the Dubinin–Radushkevich model (0.29 kJ·mol^−1^ for Pb(II) and 0.35 kJ·mol^−1^ for Ni(II)) and the free energy of adsorption from the Temkin model (0.23 kJ·mol^−1^ for Ni(II) and 0.10 kJ·mol^−1^ for Pb(II)) indicate the dominance of physical processes in the ion binding mechanism. The adsorption kinetics best correspond to the pseudo-second-order model, which suggests the dominant role of chemisorption in controlling the process rate. The analysis of the Weber and Morris intramolecular diffusion model showed two stages of adsorption, which suggests that initially the diffusion of metal ions to the biosorbent surface dominates, and in the later stages, more complex mechanisms may occur, such as intrapore diffusion or limitation of the availability of free adsorption sites. However, the adsorption efficiency depends on the appropriate adjustment of the process conditions to the specificity of the pollutant, which emphasizes the need for optimization in practical water purification applications.

In summary, *Spirulina* sp. is an effective, inexpensive, and environmentally friendly biosorbent for the removal of heavy metals from water, making it a promising alternative to conventional treatment technologies such as activated carbon adsorption or chemical processes. However, the study has certain limitations. The experiments were conducted in controlled laboratory conditions using synthetic solutions, which may not fully reflect the complexity of real wastewater. Factors such as competing ions, organic matter, and pH fluctuations could affect biosorption efficiency. Long-term stability, potential desorption under variable conditions, and performance in continuous systems were not investigated. To support practical application, further research is needed to optimize operational parameters under real wastewater scenarios, evaluate environmental safety (e.g., effluent ecotoxicity), and assess the regeneration and reuse potential of the biosorbent. Addressing these aspects will enable the sustainable use of *Spirulina* sp. in water and wastewater treatment, aligned with circular economy principles.

## Figures and Tables

**Table 1:** Single and binary system—list of adsorption models used [62,63,64,65,66,67,68,69,70,71].

Isotherm	Equation
Freundlich	$q_{e}=K_{F}\cdot {{(C}_{e})}^{\frac{1}{n}};\;\;\;\;\ln q_{e}=\frac{1}{n}lnC_{e}+lnK_{F}$
Langmuir	$q_{e}=q_{\max}\frac{K_{L}\cdot C_{e}}{1+K_{L}\cdot C_{e}};\;\;\;\;\frac{1}{q_{e}}=\frac{1}{K_{L}\cdot q_{max}\cdot C_{e}}+\frac{1}{q_{max}}$
Redlich-Peterson	$q_{e}=\frac{K_{R}\cdot C_{e}}{1+a_{R}\cdot C_{e}^{\beta }};\;\;\;\;\ln(\frac{K_{R}\cdot C_{e}}{q_{e}}-1)=\beta lnC_{e}+lna_{R}$
Jovanović	$q_{e}=q_{\max}\left(1-\exp\left(-K_{J}\cdot C_{e}\right)\right);\;\;\;\;\ln q_{e}=K_{J}\cdot C_{e}+lnq_{max}$
Halsey	$q_{e}=(\frac{K_{H}}{C_{e}}{)}^{\frac{1}{n_{H}}};\;\;\;\;\ln q_{e}=\frac{1}{n_{H}}lnK_{H}-\frac{1}{n_{H}}lnC_{e}$
Temkin	$q_{e}=B_{T}\cdot \ln{(K_{T}\cdot C}_{e});\;\;\;\;q_{e}=B_{T}\cdot \ln K_{T}+B_{T}\cdot \ln C_{e};\;\;\;\;B_{T}=\frac{RT}{b}$
Dubinin–Radushkevich	$q_{e}=q_{\max}\cdot \exp(-K_{DR}(\mathrm{RTln}(1+\frac{1}{C_{e}}{))}^{2});$ ${lnq}_{e}=-K_{DR}\cdot {ɛ}^{2}+{lnq}_{max};\;\;E=\frac{1}{{\left(2K_{DR}\right)}^{\frac{1}{2}}};\;\;ɛ=RTln(1+\frac{1}{C_{e}})$
Brunauer, Emmett, and Teller	$q_{e}=q_{\max\;}\frac{K_{BET}\frac{C_{e}}{C_{0}}}{\left(1-\frac{C_{e}}{C_{0}}\right)\left[1+\left(K_{BET}-1\right)\frac{C_{e}}{C_{0}}\right]};$ $\frac{\frac{C_{e}}{C_{0}}}{q_{e}\left(1-\frac{C_{e}}{C_{0}}\right)}=q_{\infty }\frac{K_{BET}-1}{K_{BET}{\cdot q}_{\max\infty }}\cdot \frac{C_{e}}{C_{0}}+\frac{1}{K_{BET}{\cdot q}_{\max}}$

Explanation of abbreviations: q_e_—the concentration of the adsorbate on the adsorbent surface [mg∙g^−1^], K_F_—Freundlich constant [mg^1−1/n^∙L^1/n^∙g^−1^], C_e_—denotes the ion concentration at equilibrium [mg∙L^−1^], n—heterogeneity factor, q_max_—signifies the maximal ion adsorption capacity [mg∙g^−1^], K_L_—Langmuir constant [L∙mg^−1^], K_R_—Redlich-Peterson isotherm constant [L∙g^−1^], $a_{R}$—Redlich-Peterson isotherm constant [L∙mg^−1^], β—dimensionless exponent (value ranges from 0 to 1), K_J_—Jovanović isotherm constant [L∙mg^−1^], K_H_—Halsey isotherm constant [mg^n−1^·g^−n^·L], n_H_—parameter describing the adsorption intensity, K_T_—Temkina isotherm constant [L∙g^−1^], B—constant related to adsorption energy, R—universal gas constant [8.314 J·mol^−1^·K^−1^], T—temperature [K], b—Temkin constant related to the heat of adsorption [J∙mol^−1^], K_DR_—Dubinin–Radushkevich isotherm constant [mol^2^·kJ^−2^], ε—Polanyi potential, E—energy [J∙mol^−1^], C_0_—initial concentration of the substance [mg∙L^−1^], K_BET_—adsorption constant [L^−1^∙mg].

**Table 2:** The values of isotherm adsorption parameters for removal of Ni(II) and Pb(II) by *Spirulina* sp.

Isotherm	Parameter	Ni(II)	Pb(II)
Freundlich	K_F_ [mg^1−1/n^·L^1/n^·g^−1^]	1.15	3.90
	n	2.1	2.0
	R^2^	0.9799	0.9433
Langmuir	K_L_ [L·mg^−1^]	0.12	6.4·10^−3^
	q_max_ [mg·g^−1^]	20.8	93.5
	R^2^	0.8987	0.9913
Redlich and Peterson	K_R_ [L·g^−1^]	7.3	4.3
	a_R_ [(L·mg^−1^)^β^]	54.6	16.3
	β	0.52	0.51
	R^2^	0.9833	0.9452
Jovanović	K_J_ [L·mg^−1^]	6.0·10^−4^	9.0·10^−4^
	q_max_ [mg·g^−1^]	8.2	16.4
	R^2^	0.8045	0.5719
Halsey	K_H_ [mg^n−1^·g^−n^·L]	1.5·10^−4^	2.0·10^−4^
	n_H_	0.83	1.06
	R^2^	0.9539	0.8759
Temkin	K_T_ [L·mg^−1^]	0.05	0.21
	B_T_ [J·mol^−1^]	10.5	24
	b	232	101
	R^2^	0.7438	0.9268
Dubinin and Radushkevich	K_DR_ [mol^2^·J^−2^]	4.9·10^−6^	6·10^−6^
	q_max_ [mg·g^−1^]	20.1	56.8
	E [J·mol^−1^]	353.5	288.7
	R^2^	0.4264	0.6125
Brunauer, Emmett, and Teller	K_BET_ [L·mg^−1^]	1.00	1.00
	q_max_ [mg·g^−1^]	6·10^−2^	7·10^−3^
	R^2^	0.2520	0.0595

**Table 3:** Kinetic model constants and correlation coefficients for the adsorption systems.

Kinetic Model	Parameter	Ni(II)	Pb(II)
Pseudo-first-order	k_1_ [min^−1^] R^2^	9.0·10^−4^ 0.7028	3.9·10^−3^ 0.8595
Pseudo-second-order	k_2_ [g·mg^−1^·min^−1^] R^2^	4.1·10^−4^ 0.9936	1.8·10^−2^ 0.9999
Intra-particle diffusion	k’_1_ [mg·g^−1^·min^−1/2^] b_1_ [mg·g^−1^] R^2^	0.98 3.7 0.8723	0.17 53.9 0.9979
	k’_2_ [mg·g^−1^·min^−1/2^] b_2_ [mg·g^−1^] R^2^	0.32 10.0 0.7683	0.04 56.1 0.6157

## Data Availability

The original contributions presented in this study are included in the article. Further inquiries can be directed to the corresponding authors.
